# Impact of premenstrual syndrome and premenstrual dysphoric disorder on maternal antenatal depression

**DOI:** 10.1371/journal.pone.0315628

**Published:** 2024-12-19

**Authors:** Nozomi Ihara, Yoko Sato, Subaru Ikeda, Hiromi Matsufuji, Kimiyo Kikuchi, Yoshiko Suetsugu, Naoko Hikita, Seiichi Morokuma

**Affiliations:** Department of Health Sciences, Graduate School of Medical Sciences, Kyushu University, Fukuoka, Japan; Federal University of Paraiba, BRAZIL

## Abstract

In this study, we aimed to determine the association of premenstrual syndrome (PMS) or premenstrual dysphoric disorder (PMDD) with maternal antenatal depression. This cross-sectional, online questionnaire-based observational study included 212 pregnant women between gestational ages 24 weeks and 28 weeks 6 days. PMS and PMDD were measured using the PMDD Scale, and maternal antenatal depression was evaluated using the Edinburgh Postnatal Depression Scale. Baseline characteristics, clinical information, and associated factors were also included in the questionnaire. Analyses were conducted using a binomial logistic regression model with Edinburgh Postnatal Depression Scale positivity (maternal antenatal depression) as the dependent variable. Having “PMDD” (odds ratio: 3.54 [95% confidence interval: 1.26–9.93], p = 0.02) and “PMS” (odds ratio 2.31 [1.10–4.87], p = 0.03) on the PMDD rating scale were significantly associated with maternal antenatal depression. Therefore, our results suggest that screening for a history of PMS or PMDD during the early antepartum interview may aid mental health care and prevent perinatal depression during the early stages of pregnancy.

## Introduction

Antenatal depression is a risk factor for preterm delivery, low birth weight, impaired growth among infants, decreased appetite, insomnia, and easy fatigability in mothers [1, 2]. It is also associated with a high risk of postpartum depression [3, 4]. A structured interview study by Kitamura et al. [5] reported a 5.6% prevalence of antenatal depression in Japan, making it the most prevalent mental disorder during pregnancy. A meta-analysis of perinatal depression in Japanese women by Tokumitsu et al. [6] showed a prevalence of 14.0% in the first 3–6 months of pregnancy, 16.3% from 6 months to delivery, 15.1% from 0–1 month postpartum, 11.6% from 1–2 months postpartum, 11.5% from 3–6 months postpartum, and 11.5% from 6 months to 1 year postpartum. This suggests a relatively high risk of antenatal depression postpartum. Therefore, identifying risk factors and taking preventive measures is crucial to prevent antenatal depression.

Risk factors for antenatal depression include various psychosocial factors; however, an association with changes in sex hormone levels has been reported [7]. Sex hormone changes can impact various physical disorders associated with menstruation, with premenstrual syndrome (PMS) and premenstrual dysphoric mood disorder (PMDD) being the most represented [8]. The International Classification of Diseases [9] defines PMS as "characterized by specific environmental, metabolic, or behavioral factors that occur during the luteal phase of the menstrual cycle, which leads to cyclic emotional, physical, or behavioral symptoms that interfere with an individual’s lifestyle." The American Psychiatric Association’s diagnostic criteria (DSM-5) [10] defines PMDD as “mood instability, irritability, discomfort, or anxiety that recurs during the premenstrual phase of the menstrual cycle and resolves before or immediately after the start of menstruation, occurring during most menstrual cycles in the past year.”

Estrogen and progesterone are involved in the regulation of mood-related biological systems, neural networks, and behavior; therefore, changes in their levels are thought to induce depressive symptoms [11]. A decreased level of allopregnanolone, a metabolite of progesterone, has been associated with depressive symptoms during pregnancy and the postpartum period [12, 13]. Previous studies show that allopregnanolone levels do not differ between patients with PMS and healthy individuals in the follicular phase; however, they are significantly lower in the PMS group during the luteal phase [14]. Similarly, allopregnanolone levels are reportedly lower in the PMDD group compared with healthy individuals during the luteal phase [14, 15]. These findings suggest a potential common mechanism underlying the onset of PMS, PMDD, and perinatal depression.

A systematic review of risk factors for postpartum depression by Gastaldon et al. [16] showed that the risk of developing postpartum depression was approximately double in women with a history of PMS. Sylvén et al. [17] also found a significant association between a history of PMS or PMDD and postpartum depression.

Antenatal depression may be associated with a history of PMS or PMDD; however, only a few studies have reported this. Pataky et al. [18] reported an approximately three-fold increased risk of depression at 32–42 weeks of pregnancy among individuals with a history of PMS or PMDD. Tokumitsu et al. [6] also reported a high prevalence of antenatal depression from the sixth month of pregnancy. Evidence suggests that interventions for women at risk of antenatal depression effectively prevent postnatal depression and improve pregnancy outcomes [19]. Therefore, we believe that early evaluation of at-risk individuals is necessary.

Thus, in this study, we aimed to determine the relationship between a history of PMS or PMDD and depression during the second trimester of pregnancy.

## Material and methods

### Study population

A survey recruitment page was posted on an information website for pregnant women between May 2022 and June 2022 (Milcare, Tokyo) [20]. The women surveyed from across Japan participated in the study after seeing this survey recruitment page on an information site for pregnant women. The survey participants were pregnant women aged ≥20 years with singleton pregnancies of 24–28 weeks of gestation at the time of consent. Participants were informed in advance that it would take 20 min to complete the questionnaire. The exclusion criteria were 1) psychiatric disorders, such as major depressive disorder or bipolar disorder; 2) difficulty completing the questionnaire; and 3) multiple pregnancies.

Notably, 238 respondents answered the web-based questionnaire. We excluded 26 respondents with irrelevant information. These included 13 who gave incorrect answers, as an error was observed between the expected delivery date and the current gestational weeks, and 13 who did not provide valid answers regarding their delivery history. Finally, 212 respondents were included in the analysis.

### Data collection procedures

The questionnaire included basic demographics (age, pre-pregnancy body mass index, marital status, age of partner, employment status, financial status, education history, smoking, and drinking history) and clinical information (history of childbirth, gestational age, and history of gynecological and psychiatric disorders). Factors reportedly associated with antenatal depression (desire to become pregnant, feelings at the time of pregnancy discovery, whether or not the patient lived with someone, returning to the patient’s parent’s house to give birth, whether or not someone was willing to help during pregnancy and postpartum, and sleep quality) were also assessed [21, 22].

The Edinburgh Postnatal Depression Scale (EPDS) was used to evaluate association; Cox et al. [23] developed the EPDS to quantitatively assess postpartum depression. However, a Japanese version was developed by Okano et al. [24]; it includes 10 items associated with mood depression and is scored on a four-point scale from 0 to 3. The total score ranges from 0 to 30 points, with a cut-off of 9 points. The scale was developed to screen for postpartum depression and is mainly used during the postnatal period; however, it is also currently being used to screen for antenatal depression. Usuda et al. [25] identified a cut-off score of 9 among Japanese women, validating its use from pregnancy onwards. With a cut-off score set at 13 points, the area under the curve was 0.956, sensitivity and specificity were 90.0% and 92.1%, respectively, and positive and negative predictive values were 54.5% and 98.9%, respectively. Therefore, women with an EPDS score ≥9 were considered a positive group for suspected gestational depression, and those with an EPDS score <9 were considered a negative group.

PMS and PMDD were assessed using the PMDD Rating Scale, a Japanese version of the scale developed by Miyaoka et al. [26] based on the premenstrual symptoms screening tool developed by Steiner et al. [27] using the DSM-5 PMDD diagnostic criteria. The Japanese version has a Cronbach’s alpha of 0.91, and its reliability and validity have been verified. We have also obtained permission to use the scale. The scale consists of a four-point scale (not at all: 1 point, mild: 2 points, moderate: 3 points, and severe: 4 points) that indicates whether each questionnaire item applies to PMDD, beginning 1–2 weeks before the start of menstruation and ending within a few days after the start of menstruation, during most menstrual cycles in the past year. The questionnaire consists of Item I (12 items relating to symptoms) and Item II (five items relating to difficulties at work, home, and school). The PMDD and PMS assessments were based on the premenstrual symptoms screening tool criteria developed by Steiner et al. [27], which were categorized into three groups: 1) PMDD (PMDD group), 2) moderate to severe PMS (PMS group), and 3) no or mild PMS (Non-interference group).

### Statistical analyses

χ^²^ tests were performed to assess the associations between antenatal depression and baseline characteristics, clinical information, factors reported to be associated with antenatal depression [21, 22], and the PMDD rating scale (PMDD group, PMS group, and non-interference group). Forced entry binomial logistic regression analysis was then performed using the results of the χ^²^ tests, with significance levels < 25%, as covariates to exclude variables that were evidently not significant. The IBM SPSS Statistics ver. 29 was used to perform this analysis at a 5% significance level. In addition, post hoc power analysis with alpha = 0.05 representing the statistical power was conducted using the G-power software (G-power v3.1.9.2, Universitat Kiel, Kiel, Germany).

### Ethics statement

This study was approved by the Ethics Review Committee for Clinical Research, Department of Medical Sciences, Kyushu University (approval number 22010 00).

This study was designed according to the ethical principles of the Declaration of Helsinki and followed the Ethical Guidelines for Medical and Health Research Involving Human Subjects. Written informed consent was obtained from all the participants before answering the questionnaire.

## Results

### Study population characteristics

Table 1 presents the basic characteristics of the participants analyzed. The mean age of the participants was 31.9 (standard deviation: 4.0) years. Thirty-five (16.5%) participants were underweight, 150 (70.8%) had normal weight, and 27 (12.7%) had obesity based on the non-pregnancy body mass index. Regarding employment status, 158 (74.5%) were currently employed, 23 (10.9%) left employment after the current pregnancy, and 31 (14.6%) have not been employed since before this pregnancy.

**Table 1:** EPDS scores based on attributes for participants (n = 212).

Item	(n)	%	EPDS score Mean (SD)	EPDS positive (n = 65)		EPDS negative (n = 147)		P-value
				(n)	%	(n)	%	
**Age**								
20–34 years old	156	73.6	6.8 (5.0)	47	72.3	109	74.1	0.78
≥35 years old	56	26.4	7.4 (5.5)	18	27.7	38	25.9	
**BMI at non-pregnancy**								
Thin (BMI<18.5)	35	16.5	8.1 (5.1)	15	23.1	20	13.6	0.18
Normal (18.5 ≤BMI<25)	150	70.8	6.7 (5.2)	44	67.7	106	72.1	
Overweight (BMI≥25)	27	12.7	6.8 (4.8)	6	9.2	21	14.3	
**Marital status**							
Married	212	100.0	7.0 (5.1)	65	100	147	100	―
Single/Divorced	0	0.0	―	0	0	0	0	
**Age of partner**							
20s	44	20.8	7.6 (5.7)	18	27.7	26	17.7	0.37
30s	133	62.7	6.7 (4.8)	37	57.0	96	65.3	
40s	32	15.1	7.6 (5.5)	9	13.8	23	15.6	
50s	3	1.4	5.3 (5.1)	1	1.5	2	1.4	
**Employment status**								
Currently employed (including maternity and paternity leave)	158	74.5	6.8 (5.0)	51	78.5	107	72.8	0.13
Had left employment after this pregnancy	23	10.9	9.4 (5.3)	9	13.8	14	9.5	
Had not been employed before this pregnancy	31	14.6	6.0 (5.1)	5	7.7	26	17.7	
**Financial status**								
Comfortable	22	10.4	5.3 (6.2)	6	9.2	16	10.9	0.71
Not comfortable, but satisfied	111	52.3	6.3 (4.7)	31	47.7	80	54.4	
Relatively not comfortable	67	31.6	8.1(4.7)	24	36.9	43	29.3	
Not at all comfortable	12	5.7	9.8 (6.9)	4	6.2	8	5.4	
**Education history**								
Middle school	1	0.5	3.0	0	0	1	0.7	0.57
High school	29	13.7	8.6 (6.5)	12	18.5	17	11.5	
Junior colleges and vocational schools	51	24.0	7.0 (5.0)	15	23.0	36	24.5	
College/University or more	131	61.8	6.7 (4.8)	38	58.5	93	63.3	
**Smoking history**								
Not currently smoking	210	99.1	7.0 (5.1)	64	98.5	146	99.3	0.52
Currently smoking	2	0.9	10.0 (5.7)	1	1.5	1	0.7	
**Drinking history**								
Not currently drinking	208	98.1	6.9 (5.0)	63	96.9	145	98.6	0.59
Currently drinking	4	1.9	11.3 (8.9)	2	3.1	2	1.4	
**History of childbirth**								
Primipara	130	61.3	7.4 (5.0)	44	67.7	86	58.5	0.21
Multipara	82	38.7	6.3 (5.3)	21	32.3	61	41.5	
**Gestational age**								
24–26 weeks	122	57.5	7.2 (5.0)	40	61.5	82	55.8	0.43
27–28 weeks	90	42.5	6.7 (5.2)	25	38.5	65	44.2	
**History of gynecological disorder**								
No	145	68.4	6.4 (4.8)	39	60.0	106	72.1	0.08
Yes	67	31.6	8.3 (5.5)	26	40.0	41	27.9	
**History of psychiatric disorder**								
No	197	92.9	6.6 (4.7)	55	84.6	142	96.6	**0.03**
Yes	15	7.1	12.4 (5.0)	10	15.4	5	3.4	
**Desire to become pregnant**								
Planned pregnancy (natural conception)	123	58.0	7.1 (5.4)	37	56.9	86	58.5	0.50
Planned pregnancy (fertility treatment)	44	20.8	6.1 (4.7)	11	16.9	33	22.4	
Unexpected but wanted to conceive	41	19.3	7.4 (4.6)	15	23.1	26	17.7	
Did not want to conceive	4	1.9	8.2 (5.6)	2	3.1	2	1.4	
**Feelings at the time of pregnancy discovery**								0.25
I was very happy/Unexpected, but happy	204	96.2	6.9 (5.1)	61	93.8	143	97.3	
I don’t know/ I was in trouble	8	3.8	8.1 (4.7)	4	6.2	4	2.7	
**Living with partner**								
Yes	208	98.1	6.9 (5.1)	62	95.4	146	99.3	0.09
No	4	1.9	12.8 (5.1)	3	4.6	1	0.7	
**Living with parents**								
Yes	6	2.8	10.8 (5.4)	3	4.6	3	2.0	0.37
No	206	97.2	6.9 (5.1)	62	95.4	144	98.0	
**Returning to parent’s house to give birth**								
Yes	67	31.6	7.1 (4.9)	20	30.8	47	32.0	0.33
No	138	65.1	6.7 (5.1)	41	63.0	97	66.0	
Not yet decided.	7	3.3	12.0 (5.3)	4	6.2	3	2.0	
**Whether or not someone was willing to help during pregnancy**								
Yes	211	99.5	7.0 (5.1)	65	100	146	99.3	―
No	1	0.5	5.0	0	0	1	0.7	
**Whether or not someone was willing to help in postpartum**								
Yes	209	98.6	7.0 (5.1)	65	100	144	98.0	0.55
No	3	1.4	6.3 (1.5)	0	0	3	2.0	
**Sleep qualit**y								
Good	92	43.4	5.3 (4.4)	15	23.1	77	52.4	**<0.001**
Poor	120	56.6	8.3 (5.3)	50	76.9	70	47.6	
**PMDD scale**								
PMDD	24	11.3	10.6 (6.0)	13	20.0	11	7.5	**0.001**
PMS	66	31.1	7.7 (4.3)	26	40.0	40	27.2	
Non-interference	122	57.6	5.9 (5.0)	26	40.0	96	65.3	

N.S: not significant.

EPDS, Edinburgh Postnatal Depression Scale Japanese version; BMI, Body Mass Index; PMDD, premenstrual dysphoric disorder; PMS, premenstrual syndrome.

### Descriptive statistics for each scale

Table 1 presents the descriptive statistics for each scale. The EPDS had a mean score of 7.0 (standard deviation 5.1) and a median score of 6 (range 0–23), with 65 (30.7%) and 147 (69.3%) in the positive (≥ 9 points) and negative (< 9 points) groups, respectively (Table 1). Consequently, the proportion of positives suspected of pregnancy-related depression was 30.7%.

For the PMDD rating scale, 24 (11.3%), 66 (31.1%), and 122 (57.6%) were in the PMDD, PMS, and non-interference groups, respectively (Table 1).

### EPDS scores based on participants’ attributes

EPDS scores and EPDS (positive and negative groups) based on attributes are listed in Table 1. Significant differences between the positive and negative EPDS groups were observed for the following: “presence of a history of psychiatric disorders,” “sleep quality (good, bad),” and “PMDD scale” (p < 0.05) (Table 1).

#### Association of a history of PMS or PMDD on the EPDS

Table 2 shows the effect of a history of PMS or PMDD on the EPDS (positive and negative groups). Therefore, to identify factors significantly associated with antenatal depression, a forced entry binomial logistic regression analysis was performed with EPDS (positive and negative groups) as the dependent variable and each factor below the 25% significance level in the above χ^2^ test as the independent variable. The results showed that the following factors were significantly associated with positive EPDS: “having” a history of mental illness (odds ratio [OR]: 5.05, 95% confidence interval [CI]: 1.35–18.88), “poor” sleep quality (OR: 3.39, 95% CI: 1.63–7.07), “PMDD” on the PMDD scale (OR: 3.54, 95% CI: 1.26–9.93), and “PMS” on the PMDD scale (OR: 2.31, 95% CI: 1.10–4.87). Post hoc power calculations indicated that the study sample size yielded > 80% power for the primary outcomes.

**Table 2:** Effect of a history of PMS/PMDD on EPDS.

PMDD scale	n	EPDS positive (>9 points)		Multivariable model^*^		
		n	%	OR	95%CI	P -value
Non-interference	122	26	21.3	Reference		
PMS	66	26	39.3	2.31	[1.10–4.87]	**0.03**
PMDD	24	13	54.2	3.54	[1.26–9.93]	**0.02**

^*^Logistic regression analysis.

BMI at non-pregnancy, employment status, history of childbirth, history of gynecological disorder, history of psychiatric disorder, living with a partner, and sleep quality were entered as covariates.

OR, odds ratio; CI, confidence interval.

## Discussion

The results of this study indicate a significant association between PMS and PMDD and antenatal depression.

Pataky et al. [18] reported that a history of PMS or PMDD approximately triples the risk of depression during late pregnancy (32–42 weeks), and the present study showed a similar OR of 2.31 for PMS and 3.54 for PMDD, with a different association from that in early pregnancy. This suggests a need for earlier intervention. Regarding postpartum depression, Cao et al. [11] found that changes in estrogen and progesterone can cause depressive symptoms, and women sensitive to hormonal fluctuations may develop both PMS or PMDD and postpartum depression, as the sudden drop in hormone levels occurs during the luteal phase and after birth. Yang et al. reported that using the Swedish nationwide registers, a bidirectional association was observed between premenstrual disorders and pre- and postpartum depression. These findings suggest that a history of premenstrual disorders influences susceptibility to postpartum depression and vice versa, supporting a common etiology for both disorders [28]. The decline during pregnancy is less rapid than in the postpartum period; however, a study by Fan et al. [7] investigating the association between sex hormones and psychological distress during pregnancy reported that estrogen and progesterone blood levels were negatively correlated with the development of depression.

Furthermore, lower serum allopregnanolone levels were associated with the development of depressive symptoms during late pregnancy (37–40 weeks age of gestation) [29]. Allopregnanolone has sedative and anxiolytic effects and has also been negatively associated with the development of PMS or PMDD and postpartum depression [12, 13]. A history of PMS or PMDD may affect sex hormone levels during pregnancy, resulting in depressive symptoms; however, these levels were not measured in the present study. Thus, the association between sex hormone levels in patients with PMS or PMDD during pregnancy and depression should be investigated in the future.

Evidence suggests that interventions targeting antenatal depression risk can prevent postnatal depression and improve pregnancy outcomes [19]. This observation is supported by a systematic review and meta-analysis [30]. Therefore, the early identification of prenatal depression can mitigate its incidence and associated pregnancy outcomes, underscoring the importance of routine screening, diagnosis, and timely treatment during pregnancy [31]. However, failure to address antenatal depression promptly may escalate into postpartum depression [32]. Therefore, early identification of at-risk cases during pregnancy is crucial for safeguarding mothers from postpartum depression. Furthermore, it has been reported that PMS history also affects mother-infant bonding [33]. Thus, the results of this study may help identify at-risk cases.

This study has some limitations. First, this study used the results of the EPDS to assess risk factors for antenatal depression; however, the EPDS is a screening test for perinatal depression and is not used for the final diagnosis of perinatal depression. Second, there may have been a selection bias and issues with data reliability due to the web-based nature of the survey. For example, there may have been an information bias, such as recall bias, because the survey was self-administered and included questions about past information. Third, PMDD and PMS were assessed using questionnaires rather than being diagnosed through interviews. Finally, this was a cross-sectional study, and future longitudinal studies are necessary.

In conclusion, we found that having PMDD or PMS was significantly associated with maternal antenatal depression. Our findings suggest that screening for a history of PMS or PMDD during early antenatal interviews by healthcare providers, including sexual and reproductive health practitioners, may be useful in identifying patients for mental health care services to prevent perinatal depression during early pregnancy.

## Supporting information

S1 File (PDF)

## References

[1] Räisänen S, Lehto SM, Nielsen HS, Gissler M, Kramer MR, Heinonen S. Risk factors for and perinatal outcomes of major depression during pregnancy: a population-based analysis during 2002–2010 in Finland. BMJ Open. 2014;4: e004883. doi: 10.1136/bmjopen-2014-004883 25398675 PMC4244456

[2] Jarde A, Morais M, Kingston D, Giallo R, MacQueen GM, Giglia L, et al. Neonatal outcomes in women with untreated antenatal depression compared with women without depression: a systematic review and meta-analysis. JAMA psychiatry. 2016;73: 826–837. doi: 10.1001/jamapsychiatry.2016.0934 27276520

[3] Sugishita K, Kamibeppu K. Relationship between prepartum and postpartum depression to use EPDS. Japanese Journal of Maternal Health. 2013;53: 444–450.

[4] Luciano M, Di Vincenzo M, Brandi C, Tretola L, Toricco R, Perris F, et al. Does antenatal depression predict post-partum depression and obstetric complications? Results from a longitudinal, long-term, real-world study. Front Psychiatry. 2022;13: 1082762. doi: 10.3389/fpsyt.2022.1082762 36590632 PMC9795022

[5] Kitamura T, Yoshida K, Okano T, Kinoshita K, Hayashi M, Toyoda N, et al. Multicentre prospective study of perinatal depression in Japan: incidence and correlates of antenatal and postnatal depression. Arch Womens Ment Health. 2006;9: 121–130. doi: 10.1007/s00737-006-0122-3 16547826

[6] Tokumitsu K, Sugawara N, Maruo K, Suzuki T, Shimoda K, Yasui-Furukori N. Prevalence of perinatal depression among Japanese women: a meta-analysis. Ann Gen Psychiatry. 2020;19: 1–8. 32607122 10.1186/s12991-020-00290-7 PMC7320559

[7] Fan F, Zou Y, Ma A, Yue Y, Mao W, Ma X. Hormonal changes and somatopsychologic manifestations in the first trimester of pregnancy and post partum. Int J Gynecol Obstet. 2009;105: 46–49. doi: 10.1016/j.ijgo.2008.12.001 19185297

[8] Tiranini L, Nappi RE. Recent advances in understanding/management of premenstrual dysphoric disorder/premenstrual syndrome. Fac Rev. 2022;11: 11. doi: 10.12703/r/11-11 35574174 PMC9066446

[9] World Health Organization. International Statistical Classification of Diseases and related health problems: Alphabetical index. World Health Organization; 2004.

[10] APA AP. Diagnostic and statistical manual of mental disorders. The American Psychiatric Association; 2013.

[11] Cao S, Jones M, Tooth L, Mishra G. Does premenstrual syndrome before pregnancy increase the risk of postpartum depression? Findings from the Australian Longitudinal Study on Women’s Health. J Affect Disord. 2021;279: 143–148. doi: 10.1016/j.jad.2020.09.130 33049432

[12] McEvoy K, Osborne LM. Allopregnanolone and reproductive psychiatry: an overview. Int Rev Psychiatry. 2019;31: 237–244. doi: 10.1080/09540261.2018.1553775 30701996 PMC6594874

[13] Pinna G, Almeida FB, Davis JM. Allopregnanolone in postpartum depression. Front Glob Women’s Health. 2022;3: 823616. doi: 10.3389/fgwh.2022.823616 35558166 PMC9088875

[14] Monteleone P, Luisi S, Tonetti A, Bernardi F, Genazzani AD, Luisi M, et al. Allopregnanolone concentrations and premenstrual syndrome. Eur J Endocrinol. 2000;142: 269–273. doi: 10.1530/eje.0.1420269 10700721

[15] Bičíková M, Dibbelt L, Hiill M, Hampl R, Starka L. Allopregnanolone in women with premenstrual syndrome. Horm Metab Res. 1998;30: 227–229. doi: 10.1055/s-2007-978871 9623639

[16] Gastaldon C, Solmi M, Correll CU, Barbui C, Schoretsanitis G. Risk factors of postpartum depression and depressive symptoms: umbrella review of current evidence from systematic reviews and meta-analyses of observational studies. Br Psychiatry. 2022;1: 1–2. doi: 10.1192/bjp.2021.222 35081993

[17] Sylvén SM, Ekselius L, Sundström‐Poromaa I, Skalkidou A. Premenstrual syndrome and dysphoric disorder as risk factors for postpartum depression. Acta Obstet Gynecol Scand. 2013;92: 178–184. doi: 10.1111/aogs.12041 23157487

[18] Pataky EA, Ehlert U. Longitudinal assessment of symptoms of postpartum mood disorder in women with and without a history of depression. Arch Womens Ment Health. 2020;23: 391–399. doi: 10.1007/s00737-019-00990-4 31350668

[19] Zhao Y, Munro-Kramer ML, Shi S, Wang J, Luo J. A randomized controlled trial: effects of a prenatal depression intervention on perinatal outcomes among Chinese high-risk pregnant women with medically defined complications. Arch Womens Ment Health. 2017;20: 333–344. doi: 10.1007/s00737-016-0712-7 28058505

[20] Corporation Milcare. Service. *Milcare*. Available from: http://www.milcare.co.jp/.

[21] Lancaster CA, Gold KJ, Flynn HA, Yoo H, Marcus SM, Davis MM. Risk factors for depressive symptoms during pregnancy: a systematic review. Am J Obstet Gynecol. 2010;202: 5–14. doi: 10.1016/j.ajog.2009.09.007 20096252 PMC2919747

[22] Gao M, Hu J, Yang L, Ding N, Wei X, Li L, et al. Association of sleep quality during pregnancy with stress and depression: a prospective birth cohort study in China. BMC Pregnancy Childbirth. 2019;19: 1–8. 31775666 10.1186/s12884-019-2583-1 PMC6882237

[23] Cox JL, Holden JM, Sagovsky R. Detection of postnatal depression: development of the 10-item Edinburgh Postnatal Depression Scale. Br J Psychiatry. 1987;150: 782–786. 3651732 10.1192/bjp.150.6.782

[24] Okano T. Validation and reliability of a Japanese version of the EPDS. Arch Psychiatr Diagn Clin Eval. 1996;7: 525.

[25] Usuda K, Nishi D, Okazaki E, Makino M, Sano Y. Optimal cut-off score of the Edinburgh Postnatal Depression Scale for major depressive episode during pregnancy in Japan. Psychiatry Clin Neurosci. 2017;71: 836–842. doi: 10.1111/pcn.12562 28767198

[26] Miyaoka Y, Akimoto Y, Kamo T, Ueda K. The reliability and validity of the newly developed PMDD scale. J Jp Soc Psychosom Obstet Gynecol. 2009;14: 194–201.

[27] Steiner M, Macdougall M, Brown E. The premenstrual symptoms screening tool (PSST) for clinicians. Arch Womens Ment Health. 2003;6: 203–209. doi: 10.1007/s00737-003-0018-4 12920618

[28] Yang Q, Bränn E, Bertone-Johnson ER, Sjölander A, Fang F, Oberg AS, et al. The bidirectional association between premenstrual disorders and perinatal depression: A nationwide register-based study from Sweden. PLoS Med. 2024;21: e1004363. doi: 10.1371/journal.pmed.1004363 38547436 PMC10978009

[29] Hellgren C, Åkerud H, Skalkidou A, Bäckström T, Sundström-Poromaa I. Low serum allopregnanolone is associated with symptoms of depression in late pregnancy. Neuropsychobiology. 2014;69: 147–153. doi: 10.1159/000358838 24776841

[30] Yasuma N, Narita Z, Sasaki N, Obikane E, Sekiya J, Inagawa T, et al. Antenatal psychological intervention for universal prevention of antenatal and postnatal depression: a systematic review and meta-analysis. J Affect Disord. 2020;273: 231–239. doi: 10.1016/j.jad.2020.04.063 32421608

[31] Joshi U, Lyngdoh T, Shidhaye R. A cross-sectional study on ante-natal depression in a tertiary health care hospital in Navi Mumbai. Int J Community Med Public Health. 2020;7: 599.

[32] Sheeba B, Nath A, Metgud CS, Krishna M, Venkatesh S, Vindhya J, et al. Prenatal depression and its associated risk factors among pregnant women in Bangalore: a hospital-based prevalence study. Front Public Health. 2019;7: 1–9. 31131270 10.3389/fpubh.2019.00108 PMC6509237

[33] Yücesoy H, Erbil N. Relationship of premenstrual syndrome with postpartum depression and mother–infant bonding. Perspect Psychiatr Care. 2022;58: 1112–1120. doi: 10.1111/ppc.12909 34231233

