# Supplementary material for: Impact of premenstrual syndrome and premenstrual dysphoric disorder on maternal antenatal depression
Source: PLoS One. 2024 Dec 19;19(12):e0315628. doi: 10.1371/journal.pone.0315628 (PMC11658527; doi:10.1371/journal.pone.0315628)
Supplement: S1 File — (PDF) [file pone.0315628.s001.pdf]

interference	PMS	PMDD	EPDS P=1
1	0	0	0
0	1	0	0
1	0	0	0
0	0	1	0
1	0	0	0
0	0	1	1
0	0	1	0
0	1	0	0
1	0	0	0
0	1	0	0
0	1	0	0
1	0	0	0
0	1	0	0
0	1	0	0
0	0	1	1
0	1	0	0
1	0	0	0
0	1	0	1
1	0	0	0
1	0	0	0
1	0	0	0
1	0	0	0
0	1	0	0
0	1	0	1
0	1	0	0
0	1	0	1
0	0	1	0
1	0	0	0
1	0	0	0
1	0	0	1
1	0	0	0
0	1	0	1
0	0	1	0
1	0	0	0
0	0	1	0
0	0	1	1
1	0	0	0
0	1	0	0

1	0	0	0
1	0	0	0
1	0	0	1
0	0	1	0
1	0	0	1
0	1	0	0
0	1	0	0
1	0	0	0
1	0	0	1
1	0	0	0
1	0	0	0
1	0	0	1
1	0	0	1
0	1	0	0
1	0	0	0
1	0	0	1
1	0	0	0
0	1	0	0
0	1	0	0
1	0	0	0
0	0	1	1
0	1	0	0
1	0	0	0
0	1	0	1
0	1	0	1
1	0	0	1
0	0	1	1
1	0	0	0
0	1	0	1
0	0	1	0
1	0	0	1
0	1	0	1
0	1	0	0
0	0	1	1
1	0	0	0
1	0	0	0
0	1	0	1
1	0	0	1
1	0	0	0

1	0	0	0
1	0	0	0
1	0	0	0
1	0	0	1
1	0	0	0
1	0	0	1
1	0	0	0
1	0	0	0
0	1	0	0
1	0	0	0
0	1	0	1
1	0	0	0
1	0	0	1
1	0	0	1
0	1	0	0
1	0	0	0
1	0	0	0
1	0	0	0
0	1	0	0
1	0	0	0
1	0	0	1
0	1	0	1
1	0	0	0
0	1	0	0
0	1	0	1
0	0	1	1
0	1	0	0
1	0	0	0
1	0	0	0
1	0	0	0
1	0	0	0
1	0	0	0
1	0	0	0
0	0	1	1
1	0	0	0
1	0	0	0
1	0	0	0
0	1	0	1
1	0	0	0

1	0	0	1
0	1	0	1
0	1	0	0
0	0	1	1
1	0	0	0
0	1	0	0
0	1	0	0
0	0	1	1
0	1	0	1
1	0	0	1
1	0	0	0
1	0	0	0
1	0	0	1
0	1	0	0
1	0	0	1
0	1	0	0
1	0	0	1
0	1	0	1
1	0	0	0
0	1	0	0
1	0	0	0
1	0	0	0
0	1	0	0
1	0	0	0
1	0	0	0
0	0	1	0
1	0	0	0
0	1	0	0
1	0	0	1
1	0	0	0
1	0	0	0
1	0	0	0
1	0	0	0
1	0	0	0
1	0	0	0
1	0	0	0
0	0	1	1
0	1	0	1
1	0	0	1

0	1	0	1
0	0	1	1
0	1	0	1
0	1	0	0
1	0	0	0
1	0	0	0
1	0	0	0
0	1	0	1
0	1	0	1
1	0	0	0
0	1	0	0
1	0	0	1
1	0	0	0
1	0	0	0
1	0	0	0
0	1	0	0
1	0	0	0
1	0	0	0
1	0	0	0
1	0	0	0
1	0	0	0
0	1	0	0
1	0	0	0
1	0	0	0
1	0	0	0
1	0	0	0
1	0	0	0
1	0	0	0
1	0	0	1
0	1	0	0
0	1	0	1
0	0	1	1
1	0	0	0
1	0	0	1
0	1	0	1
0	1	0	0
1	0	0	0
1	0	0	0
0	0	1	0

1	0	0	0
0	0	1	0
0	1	0	0
1	0	0	0
0	0	1	0
1	0	0	0
0	1	0	1
0	1	0	0
0	1	0	0
0	1	0	0
1	0	0	0
1	0	0	0
1	0	0	1
1	0	0	0
0	1	0	1
0	1	0	1
1	0	0	0
1	0	0	0
